# Unique Astrocyte Cytoskeletal and Nuclear Morphology in a Three-Dimensional Tissue-Engineered Rostral Migratory Stream

**DOI:** 10.3390/neuroglia3010003

**Published:** 2022-03-06

**Authors:** Erin M. Purvis, John C. O’Donnell, D. Kacy Cullen

**Affiliations:** 1 Center for Brain Injury and Repair, Department of Neurosurgery, Perelman School of Medicine, University of Pennsylvania, 105E Hayden Hall/3320 Smith Walk, Philadelphia, PA 19104, USA; 2 Center for Neurotrauma, Neurodegeneration and Restoration, Corporal Michael J. Crescenz Veterans Affairs Medical Center, A517 Building 21, 3900 Woodland Avenue, Philadelphia, PA 19104, USA; 3 Department of Neuroscience, Perelman School of Medicine, University of Pennsylvania, 415 Curie Blvd, Philadelphia, PA 19104, USA; 4 Department of Bioengineering, School of Engineering and Applied Science, University of Pennsylvania, 210 South 33rd Street, Suite 240 Skirkanich Hall, Philadelphia, PA 19104, USA

**Keywords:** rostral migratory stream, astrocyte, tissue engineering, nucleus, cytoskeleton, cell morphology, nuclear morphology

## Abstract

Neural precursor cells (NPCs) are generated in the subventricular zone (SVZ) and travel through the rostral migratory stream (RMS) to replace olfactory bulb interneurons in the brains of most adult mammals. Following brain injury, SVZ-derived NPCs can divert from the RMS and migrate toward injured brain regions but arrive in numbers too low to promote functional recovery without experimental intervention. Our lab has biofabricated a “living scaffold” that replicates the structural and functional features of the endogenous RMS. This tissue-engineered rostral migratory stream (TE-RMS) is a new regenerative medicine strategy designed to facilitate stable and sustained NPC delivery into neuron-deficient brain regions following brain injury or neurodegenerative disease and an in vitro tool to investigate the mechanisms of neuronal migration and cell–cell communication. We have previously shown that the TE-RMS replicates the basic structure and protein expression of the endogenous RMS and can direct immature neuronal migration in vitro and in vivo. Here, we further describe profound morphological changes that occur following precise physical manipulation and subsequent self-assembly of astrocytes into the TE-RMS, including significant cytoskeletal rearrangement and nuclear elongation. The unique cytoskeletal and nuclear architecture of TE-RMS astrocytes mimics astrocytes in the endogenous rat RMS. Advanced imaging techniques reveal the unique morphology of TE-RMS cells that has yet to be described of astrocytes in vitro. The TE-RMS offers a novel platform to elucidate astrocyte cytoskeletal and nuclear dynamics and their relationship to cell behavior and function.

## 1. Introduction

Neurogenesis, or the birth of new neurons in the brain, continues in the subventricular zone (SVZ) of most mammals throughout adulthood [1,2]. Neural precursor cells (NPCs) in the SVZ of rodents can differentiate into neuroblasts and migrate through a pathway of aligned astrocytes known as the rostral migratory stream (RMS) to the olfactory bulb (OB), where they mature into functional granule, periglomerular, or glutamatergic interneurons and integrate into existing circuitry [3–6]. Following brain injury, SVZ neurogenesis is up-regulated [7–9] and neuroblasts can divert from the SVZ-RMS-OB pathway, migrate toward injured brain regions, and mature into functional, phenotypically relevant neurons [10–12]. Further, experimentally enhancing the redirection of neuroblasts from the SVZ into injured regions can induce functional recovery following injury [10,12–22].

A variety of biomaterial and tissue engineering strategies have been developed to enhance the redirection of SVZ neuroblasts throughout the brain following neuronal injury [23–25]. These strategies have increased the quantity of neuroblasts entering injured brain regions by augmenting chemoattractant cues and/or by providing a physical substrate for migration [23]. Drawing inspiration from the SVZ-RMS-OB pathway, our laboratory has developed a biologically relevant, implantable “living scaffold” that emulates the glial tube of the endogenous RMS [26–29]. This tissue-engineered rostral migratory stream (TE-RMS) is designed to promote the sustained delivery of endogenous NPCs into injured brain regions by replicating and expanding upon the endogenous RMS, which is one of the brain’s intrinsic mechanisms for neuronal replacement. Previous experiments not presented herein have demonstrated that the TE-RMS can be reliably fabricated from sources of rat and human astrocytes, mimics increased expression of key functional proteins of the endogenous rat RMS, can redirect immature rat neurons in vitro, and when implanted into the rat brain can facilitate migration of endogenous neuroblasts out of the RMS and throughout the TE-RMS [29].

**Figure 1.**
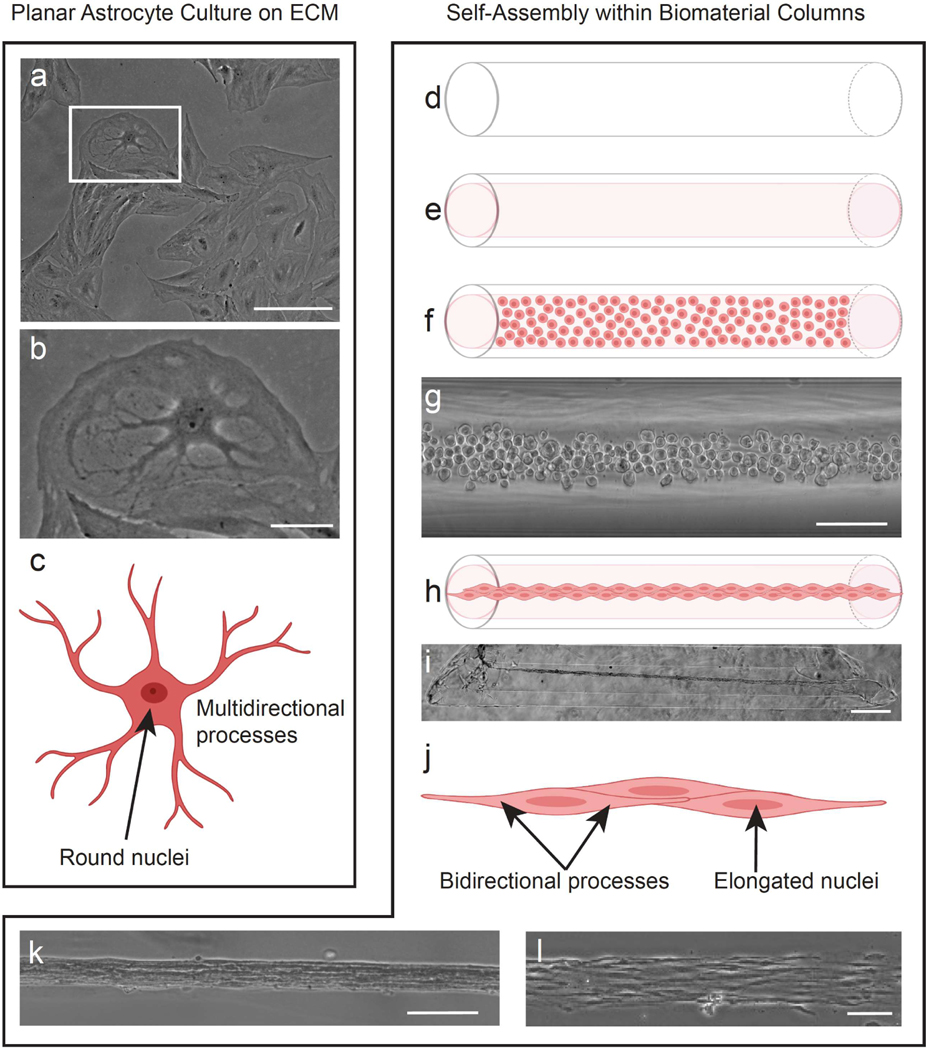
Tissue-engineered rostral migratory stream astrocytes possess unique cellular morphology compared to planar astrocytes. Phase microscope image of planar astrocytes in culture (**a**) with magnified view depicting planar astrocyte morphology in higher detail (**b**). Planar astrocytes possess round nuclei and have multidirectional processes that extend radially around the cell (**c**). TE-RMSs are fabricated in hollow agarose microcolumns (**d**) that are loaded with collagen (**e**) and then following collagen polymerization are seeded with a concentrated astrocyte suspension (**f**,**g**). Astrocytes pull the polymerized collagen off the inner lumen of the microcolumn and use it to align themselves in longitudinal bundles tethered to either end of the microcolumn (**h**,**i**). TE-RMS astrocytes possess elongated nuclei and bidirectional processes that preferentially extend in parallel with the microcolumn (**j**). Magnified phase images depict these longitudinally aligned bundles of astrocytes exhibiting this unique morphology (**k**,**l**). Scale bars: 200 microns (**a**,**g**,**k**), 50 microns (**b**), 500 microns (**i**), 100 microns (**l**).

The TE-RMS is fabricated from living astrocytes and extracellular matrix (ECM) in small diameter agarose microcolumns [27] (Figure 1). Microcolumns (Figure 1d) are cut at an angle, seeded with ECM (collagen) (Figure 1e), and then following ECM polymerization are seeded with a concentrated cell suspension of living astrocytes (Figure 1f,g). Over a short period of only 8 h, astrocytes pull the ECM off the inner lumen of the column and “zipper” themselves together into longitudinally aligned bundles of astrocytes tethered to either end of the microcolumn (Figure 1h,i). This precise mechanical manipulation of astrocytes into three-dimensional (3D) conditions forms the basic structure of the TE-RMS. The unique biomaterial conditions that mechanically manipulate astrocytes from a planar/two-dimensional (2D) culture into the 3D TE-RMS induce profound morphological changes in these cells. For instance, in previous experiments we have observed that TE-RMS astrocytes possess bipolar processes, morphological features which drastically differ from the multidirectional processes possessed by astrocytes in planar culture [26]. These morphological changes can be attributed to the specific topographic and physical features of the microcolumns that induce astrocyte self-assembly and TE-RMS formation.

Considering the important connection of cellular morphology and function, here we report a thorough morphological characterization of the changes in intermediate filament arrangement and nuclear shape between astrocytes that comprise the 3D TE-RMS compared to planar astrocyte sister cultures. We demonstrate that the nuclear elongation and precise angulation of intermediate filaments that is unique to TE-RMS astrocytes mimics that of astrocytes in the endogenous RMS compared to surrounding protoplasmic astrocytes in the rat brain. Additionally, we support these findings with high magnification and ultrastructural imaging to provide a more detailed look at this unique astrocyte phenotype in vitro. These analyses highlight the TE-RMS as a novel platform to investigate the relationship between cytoskeletal arrangement, nuclear morphology, and astrocyte cell function—further expanding the applications of this unique technology.

## 2. Materials and Methods

### 2.1. Astrocyte Cell Culture

All procedures described in this manuscript adhered to the National Institutes of Health Guide for the Care and Use of Laboratory Animals and were approved by the Institutional Animal Care and Use Committee at the University of Pennsylvania. Primary cortical astrocytes were harvested from Sprague–Dawley rat pups at postnatal day 0–1. Following dissociation (described previously [27]), astrocytes were cultured in DMEM/F12 medium supplemented with 10% Fetal Bovine Serum and 1% Penicillin-Streptomycin antibiotics in a cell culture incubator maintained at 37 °C and 5% CO_2_. Astrocytes were passaged at 80% confluency. Astrocytes between passages 4–10 were utilized for fabrication of TE-RMSs and planar astrocyte cultures.

### 2.2. Fabrication of Hydrogel Micro-Columns

TE-RMSs were fabricated in hydrogel micro-columns made of 3% agarose. Fabrication of micro-columns has been described previously [27,30,31]. Briefly, warm agarose dissolved in Dulbecoo’s phosphate-buffered saline (DPBS) was drawn into a capillary tube (inner diameter = 701 microns) with a needle (diameter = 300 microns) in the center. Following agarose cooling and capillary tube removal, the micro-column was pushed off the needle into PBS, effectively creating micro-columns with an inner diameter of 300 microns and an outer diameter of 701 microns. Previous experiments have determined these dimensions to be optimal for astrocyte bundling and TE-RMS formation [26].

### 2.3. Fabrication of Tissue-Engineered Rostral Migratory Streams and Planar Astrocyte Cultures

Fabrication of TE-RMSs has been recently described [29]. Micro-columns were cut to a 4 mm length and the inner lumen was seeded with 1 mg/mL rat tail type 1 collagen (Corning Catalog # 354236) diluted in in serum free co-culture media consisting of Neurobasal medium supplemented with 2% B27, 1% G-5, 0.25% L-glutamine, and 1% penicillin-streptomycin. Columns were placed in an incubator at 37 °C and 5% CO_2_ for around 3 h until collagen completely polymerized to coat the inner walls of the micro-columns. During the collagen polymerization period, a flask of 80% confluent astrocytes was passaged and cells were resuspended in co-culture media. Following complete collagen polymerization, micro-columns were seeded with astrocytes in co-culture media at a density of around 1 million cells/mL. Columns were then returned to the incubator at 37 °C and 5% CO_2_ and one hour later the outer edges of the columns were reinforced with 1mg/mL collagen. Columns were incubated for another hour at 37 °C and 5% CO_2_, dishes were flooded with warm co-culture media, and then columns were returned to the incubator at 37 °C and 5% CO_2_. Under these conditions, astrocytes bundle together with collagen and self-assemble into cords of longitudinally aligned astrocytes with bidirectional processes to form TE-RMSs. All experiments herein investigated TE-RMSs at 24 h following astrocyte seeding into microcolumns. For planar astrocyte samples, a flask of 80% confluent astrocytes was passaged, cells were resuspended in co-culture media, and were plated on top of polymerized 1 mg/mL collagen in a 12-well plate at a density of around 1 million cells/mL in co-culture media. For 100× fluorescent imaging of planar astrocytes, cells were cultured in 12-well plates on top of poly-L-lysine (PLL) and collagen-coated glass coverslips. For scanning electron microscopy (SEM) imaging, planar astrocytes were cultured in 24-well plates on top of PLL-coated glass coverslips.

### 2.4. Immunocytochemistry

Following TE-RMS formation, constructs were extracted from micro-columns onto poly-l-lysine glass slides for immunocytochemical analyses as previously described [29]. Planar astrocyte cultures and extracted TE-RMSs were fixed with 4% paraformaldehyde for 30 min at room temperature. Following rinsing with PBS, cultures and constructs were permeabilized with 0.3% Triton X-100 and blocked with 4% normal horse serum for one hour at room temperature and again rinsed with PBS. Cultures and constructs were incubated in the primary antibody goat anti-glial fibrillary acidic protein (GFAP) (1:1000) (Abcam Cat # ab53554, RRID: AB_880202) overnight at 4 °C. Cultures were then rinsed and incubated in the Alexa secondary antibody donkey anti-goat 568 (1:500) (Thermo Fisher Scientific Catalog #: A-11057, RRID: AB_2534104) and Hoechst solution (1:1000) (Invitrogen H3570) in the dark for two hours at 37 °C.

### 2.5. Immunohistochemistry

Five adult Sprague-Dawley rat brains from an archival tissue bank were selected for analysis of the endogenous RMS. Brains were formalin-fixed paraffin-embedded (FFPE) and sagittally sliced into 8-micron thick sections and mounted onto slides. Slides were deparaffinized, rehydrated, and placed in Tris-EDTA for heat-induced epitope retrieval. Slides were blocked in normal horse serum for 30 min and the primary antibody goat anti-GFAP (1:1000) (Abcam Cat # ab53554, RRID: AB_880202) was diluted in 1× Optimax buffer and applied overnight at 4 °C. Slides were washed in PBS Tween and incubated in the dark with the Alexa secondary antibody donkey anti-goat 568 (1:500) (Thermo Fisher Scientific Catalog #: A-11057, RRID: AB_2534104). Slides were then rinsed and incubated in Hoechst solution (1:10,000) (Invitrogen H3570) for 5 min and then rinsed again. Cover slips were mounted with fluoromount G and stored at 4 °C.

### 2.6. Imaging

Fluorescent images of cultures, constructs, and brain slices were obtained using a Nikon A1Rsi Laser Scanning Confocal microscope with a ×10, ×20, ×60, or ×100 objective (CFI Plan Apo Lambda ×10, n.a. 0.45; ×20, n.a. 0.75; ×60 Oil, n.a. 1.40; ×100, n.a. 1.45). Scanning electron microscope experiments were carried out at the CDB Microscopy Core (Perelman School of Medicine, University of Pennsylvania). Samples were washed three times with 50 mM Na-cacodylate buffer, fixed for 2 h with 2% glutaraldehyde in 50 mM Na-cacodylate buffer (pH 7.3), and dehydrated in a graded series of ethanol concentrations through 100% over a period of 2.5 h. Dehydration in 100% ethanol was done three times. Dehydrated samples were incubated for 20 min in 50% HMDS in ethanol followed by three changes of 100% HMDS (Sigma-Aldrich Co.) and followed by overnight air-drying as described previously [32]. Then samples were mounted on studs and sputter coated with gold palladium. Specimens were observed and photographed using a Quanta 250 FEG scanning electron microscope (FEI, Hillsboro, OR, USA) at 10 kV accelerating voltage.

### 2.7. Imaging Analyses, Statistics, and Reproducibility

**Figure 2.**
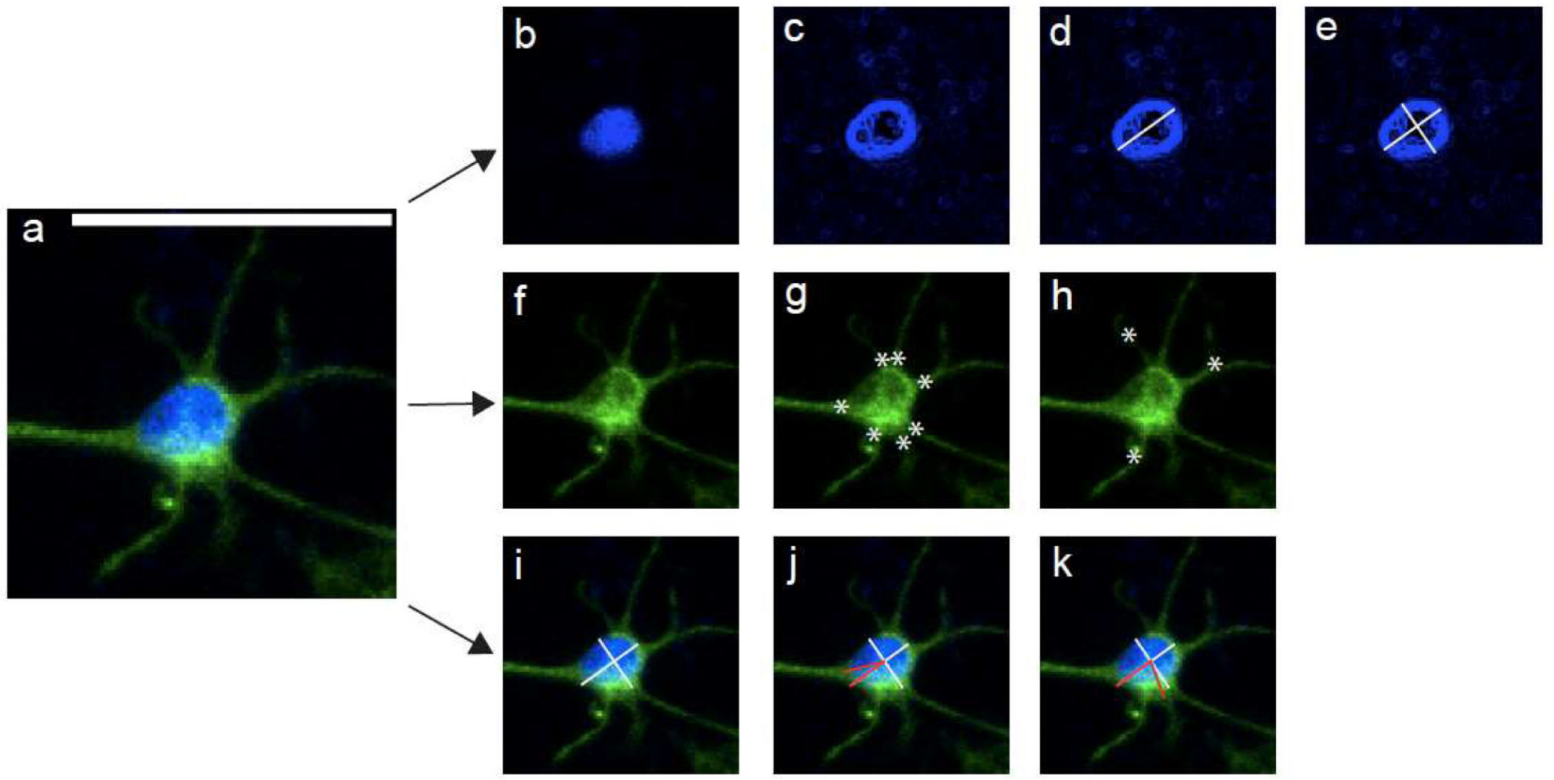
Quantification of nuclear and cytoskeletal measurements. Representative fluorescent image of single planar astrocyte (**a**) depicting nuclear (Hoechst, blue) and cytoskeleton (GFAP, green) labels. To perform nuclear length measurements, Hoechst channel was isolated (**b**) and FIJI “find edges” function was applied (**c**). To measure the long nuclear axis, a line was drawn across the longest distance from one edge of the nucleus to another (**d**). To measure the short nuclear axis, a line was drawn from one edge of the nucleus to the other and perpendicular to the long nuclear axis (**e**). GFAP channel was isolated (**f**). Main processes were GFAP extensions arising directly from the nucleus (**g**) and branch points were GFAP extensions arising from other GFAP extensions (**h**). To perform angle measurements, long and short nuclear axes were viewed in image depicting Hoechst and GFAP channels (**i**). An angle was drawn with the first point of the angle placed on the cell process (at the point where the process contacted the cell body), the second point (middle) of the angle was placed on the intersection of the cell’s long and short nuclear axes, and the third point of the angle placed on the end of the long nuclear axis (the end closer to the cell process) (**j**,**k**). Scale bar: 50 microns (**a**). *: main processes (**g**) and branch points (**h**).

Image analyses were performed using FIJI (Fiji Is Just ImageJ) software [33]. Statistical testing was performed in GraphPad Prism 9 for macOS. 20× fluorescent confocal images were utilized for quantification of all nuclear and cytoskeletal measurements. Fluorescent images were imported into FIJI as Nikon nd2 files, split into two channels (Hoechst and GFAP), z-stacked and compressed, and underwent background subtraction (rolling ball method; 50 pixel diameter). In in vitro planar astrocyte and TE-RMS images, all cells with non-overlapping nuclei were quantified. Across TE-RMS samples, we estimate that about 50% of TE-RMS astrocytes were excluded from analysis due to the appearance of overlapping nuclei. In in vivo images, protoplasmic and RMS astrocytes were quantified only if they possessed visible, non-overlapping nuclei and clear GFAP process extensions. Endogenous RMS astrocytes were identified within the dense, distinct meshwork of GFAP processes extending from the lateral ventricle to the olfactory bulb, as reliably identified previously by our laboratory and others [29,34]. Protoplasmic astrocytes were identified as astrocytes outside of this distinct structure. To measure the long and short nuclear axes (Figure 2a–e), the “find edges” FIJI function was applied to the Hoechst fluorescent channel. A cell’s long nuclear axis was measured as the longest distance from the one edge of the nucleus to the other. A cell’s short nuclear axis was measured from one edge of the nucleus to the other and perpendicular to the cell’s long nuclear axis. The nuclear aspect ratio of each cell was found by dividing the long nuclear axis by the short nuclear axis. In the merged fluorescent image (GFAP + Hoechst channels), the number of main processes, number of branch points, and angle of each main process were quantified for each cell. Main processes (Figure 2g) were defined as extensions of GFAP that arose directly from the cell’s nucleus. Branch points (Figure 2h) were defined as extensions of GFAP that arose from other GFAP extensions. The angle of each main process (Figure 2i–k) was measured as it deviated from the long nuclear axis. To perform angle measurements, an angle was drawn with the first point of the angle placed on the cell process (at the point where the process contacted the cell body), the second point (middle) of the angle was placed on the intersection of the cell’s long and short nuclear axes, and the third point of the angle placed on the end of the long nuclear axis (the end closer to the cell process). All angle measurements fell between 0 and 90 degrees, as 90 degrees was the maximum angle that could exist between the process and the long nuclear axis. All measurements for each cell were recorded and saved as ROIs in FIJI. For planar astrocyte cultures, 225 cells across 6 samples were quantified. For TE-RMS cultures, 275 cells across 9 samples were quantified. For in vivo samples (*n* = 5 animals), 124 protoplasmic astrocytes and 131 RMS astrocytes were quantified. Nuclear aspect ratios, number of main processes, and number of branch points (planar versus TE-RMS; protoplasmic versus RMS) were compared by two-tailed nested *t*-tests, with values for individual cells nested within each culture. For angle measurements, the median angle of each cell was selected and these values (planar versus TE-RMS; protoplasmic versus RMS) were then compared by two-tailed nested *t*-tests.

## 3. Results

### 3.1. Planar Astrocytes Have a More Complex and Varied Cytoskeletal Arrangement Compared to TE-RMS Astrocytes

**Figure 3.**
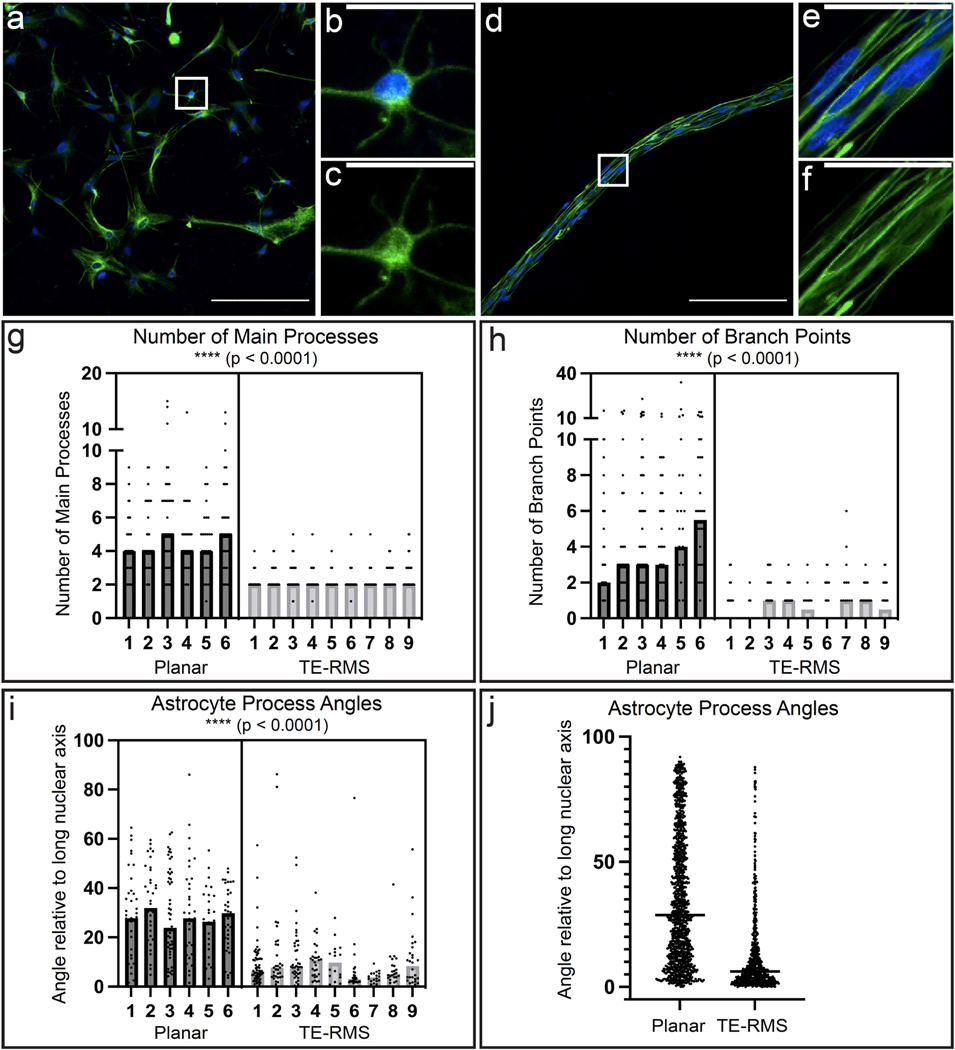
TE-RMS astrocytes exhibit distinct cytoskeletal architecture compared to planar astrocytes. Representative 20× fluorescent image of planar astrocyte culture (**a**) with zoom-in on one single astrocyte depicting nuclear (Hoechst, blue) and cytoskeleton (GFAP, green) labels (**b**) and just the cytoskeleton label (**c**). Representative 20× fluorescent image of TE-RMS (**d**) with zoom-in on one single astrocyte depicting nuclear (Hoechst) and cytoskeleton (GFAP) labels (**e**) and just the cytoskeleton label (**f**). Nested *t*-test analysis of number of main cell processes in independent planar (*n* = 6) versus TE-RMS (*n* = 9) cultures (**g**). Each point represents a single cell and bars represent the median of each group. Nested *t*-test analysis of number of branch points in independent planar and TE-RMS cultures (**h**). Each point represents a single cell and bars represent the median of each group. The angle of each main process was measured as it deviates from the long nuclear axis. Nested *t*-test analysis with each point representing the median angle from each cell and the graph bars representing the median of each group (**i**). All quantified angles (*n* = 1069 planar; *n* = 644 TE-RMS) (**j**). **** *p* < 0.0001. Scale bars: 200 microns (**a**,**d**), 50 microns (**b**,**c**,**e**,**f**).

We used fluorescence immunocytochemistry (ICC) to label nuclei (Hoechst) and the intermediate filament protein glial fibrillary acidic protein (GFAP) in planar astrocyte (*n* = 6) and TE-RMS (*n* = 9) samples to analyze the overall complexity of the cytoskeletal arrangement of in vitro cultures. For each sample, a single 20× fluorescent confocal image was used for quantification. We quantified the number of main processes, number of branch points, and angle of each main process across planar astrocytes (*n* = 225 cells) and TE-RMS astrocytes (*n* = 275 cells). Example representative images of planar (Figure 3a) and TE-RMS (Figure 3d) samples highlight the distinct cytoskeletal arrangements observed between these two in vitro groups. All cells with non-overlapping nuclei were quantified. Comparing the number of main cell processes (defined as GFAP extensions protruding directly from the cell body) by two-tailed, nested *t*-test revealed that planar astrocytes had a significantly higher number of main processes compared to TE-RMS astrocytes (Figure 3g; *t* = 10.36, df = 13, *p* < 0.0001). Number of branch points (defined as GFAP extensions protruding from another GFAP extension) were also compared by two-tailed, nested *t*-test and revealed that planar astrocytes had a significantly higher number of branch points compared to TE-RMS astrocytes (Figure 3h; *t* = 8.801, df = 14; *p* < 0.0001). Planar astrocytes demonstrated high variability across main process and branch point measurements, highlighting the heterogeneity in planar astrocyte morphology. This variability is visibly reduced in TE-RMS astrocytes which exhibit a more uniform morphology. The angle of each main process was then measured relative to the long nuclear axis of the cell. Almost all analyzed cells had more than one main process and therefore more than one angular measurement. Since these measurements did not adhere to a Gaussian distribution, the median angle for each cell was selected for statistical analyses. These values were compared via two-tailed, nested *t*-test which revealed that the main processes of planar astrocytes had larger angles compared to the main processes of TE-RMS astrocytes (Figure 3i; *t* = 13.98, df = 13, *p* < 0.0001). All measured angles for both groups (*n* = 1069 planar; *n* = 644 TE-RMS) are also presented together (Figure 3j) to display the apparent differences between planar and TE-RMS samples (statistical testing was not performed on Figure 3j as the more appropriate arrangement for given assumptions was determined *a priori* to be the nested *t*-tests as shown previously). Whereas planar astrocyte process angles were fairly evenly spread between a 0 and 90 degree deviation from the long nuclear axis, TE-RMS astrocyte process angles clustered closer to 0 degrees with far fewer angles measuring close to a 90 degree deviation. The larger the angle measurement, the further the main process deviates from the long nuclear axis. Whereas planar astrocytes generally extended processes in all directions (Figure 3b,c), TE-RMS astrocytes generally extended “bidirectional” processes in only two directions parallel with the long nuclear axis of the cell (Figure 3e,f).

### 3.2. Endogenous Protoplasmic Astrocytes Have a More Complex and Varied Cytoskeletal Arrangement Compared to Endogenous RMS Astrocytes in Rat Brain

**Figure 4.**
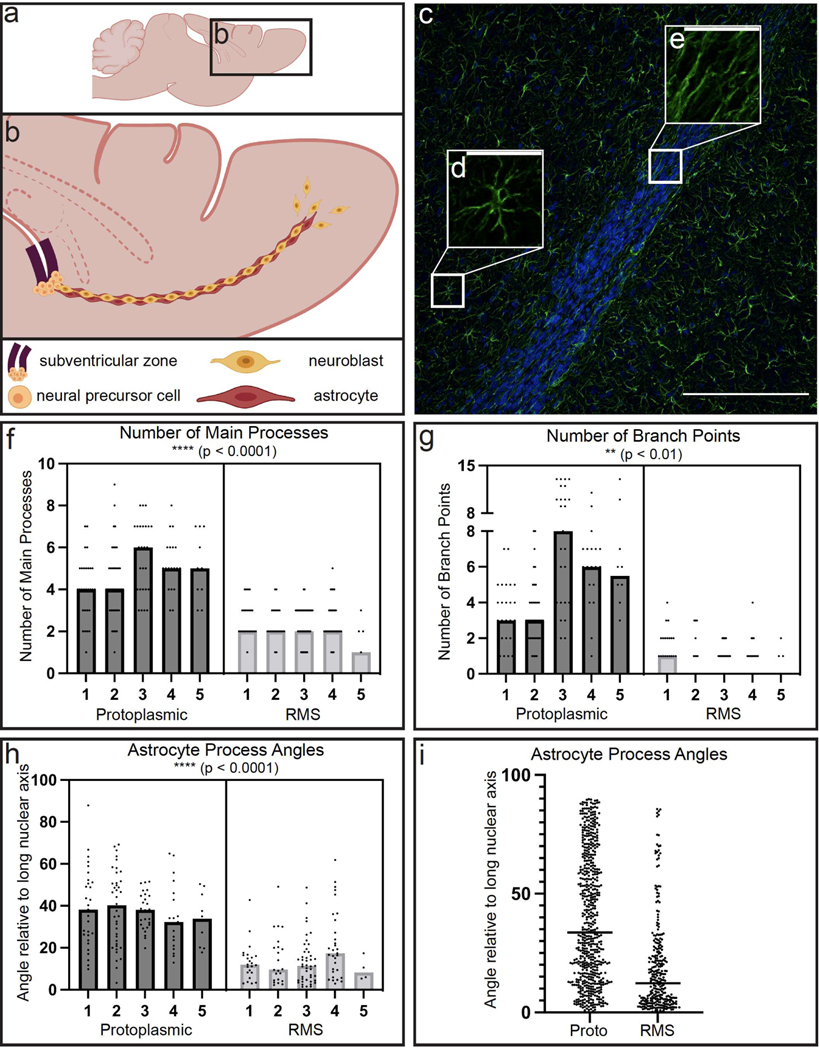
Endogenous RMS astrocytes exhibit distinct cytoskeletal architecture compared to surrounding protoplasmic astrocytes. Schematic depicting sagittal rat brain slice (**a**) and the subventricular zone-rostral migratory stream-olfactory bulb pathway (**b**). Representative fluorescent image of a sagittal rat brain slice with nuclear Hoechst stain (blue) and immunostaining for GFAP (green) at 20× magnification (**c**). Call out boxes highlight the cytoskeleton of a protoplasmic astrocyte (**d**) and RMS astrocytes (**e**). Nested *t*-test analysis of the number of main cell process possessed by protoplasmic versus RMS astrocytes (n = 5, within-subjects) (**f**). Each point represents a single cell and bars represent the median of each group. Nested *t*-test analysis of the number of branch points possessed by protoplasmic and RMS astrocytes (**g**). Each point represents a single cell and bars represent the median of each group. The angle of each main process was measured as it deviates from the long nuclear axis. Nested *t*-test analysis with each point representing the median angle from each cell and the graph bars representing the median of each group (**h**). Each point represents the median of each cell and bars represent the median of each group. All quantified angles (*n* = 585 protoplasmic; *n* = 303 RMS) (**i**). **** *p* < 0.0001, ** *p* < 0.01. Scale bars: 200 microns (**c**), 40 microns (**d**,**e**).

To compare our in vitro cytoskeletal findings to the rat brain in vivo, we applied fluorescence immunohistochemistry (IHC) techniques to label GFAP and Hoechst in sagittally sectioned (Figure 4a) adult rat brains (*n* = 5). For each subject, a single 20× fluorescent confocal image that contained RMS and non-RMS tissue was used for quantification. Like our in vitro measurements, we quantified the number of main processes, number of branch points, and angle of each main process across protoplasmic (non-RMS) astrocytes (*n* = 124 cells) and RMS astrocytes (*n* = 131 cells). All cells with visible, non-overlapping nuclei and clear GFAP process extensions within each image were analyzed. An example representative image (Figure 4c) depicting the cytoskeleton of a single protoplasmic astrocyte (Figure 4d) and several RMS astrocytes (Figure 4e) reveal distinct cytoskeletal arrangement between these two cell populations. The number of main processes were compared by a two-tailed, nested *t*-test which revealed that protoplasmic astrocytes had a significantly higher number of main processes compared to RMS astrocytes (Figure 4f; *t* = 7.334, df = 8, *p* < 0.0001). The number of branch points were also compared by two-tailed, nested *t*-test which revealed that protoplasmic astrocytes had a significantly higher number of branch points compared to RMS astrocytes (Figure 4g; *t* = 4.732, df = 8, *p* = 0.0015). The angle of each main process was again measured relative to the long nuclear axis of the cell, and the median angle measurement for each cell was compared via two-tailed, nested *t*-test which revealed that the main processes of protoplasmic astrocytes had larger angles compared to the main processes of RMS astrocytes (Figure 4h; *t* = 10.09, df = 8, *p* < 0.0001). All measured angles (*n* = 585 protoplasmic; *n* = 303 RMS) were again displayed (Figure 4i) to depict apparent differences between groups, and statistical analyses were not run on these values. The angles of protoplasmic astrocytes were evenly spread between 0 and 90 degrees, whereas the angles of RMS astrocytes clustered closer to 0 degrees. These data collectively demonstrate that endogenous protoplasmic astrocytes possess increased cytoskeletal complexity compared to astrocytes in the endogenous RMS. The increased cytoskeletal complexity of endogenous protoplasmic astrocytes compared to RMS astrocytes (Figure 4) mirrors the increased cytoskeletal complexity of planar astrocytes compared to TE-RMS astrocytes (Figure 3). In this way, the mechanical manipulation of astrocytes into the TE-RMSs caused their cytoskeletal complexity to mimic that of the endogenous rat RMS.

### 3.3. TE-RMS Astrocytes Possess Elongated Nuclei Compared to Planar Astrocyte Sister Cultures

**Figure 5.**
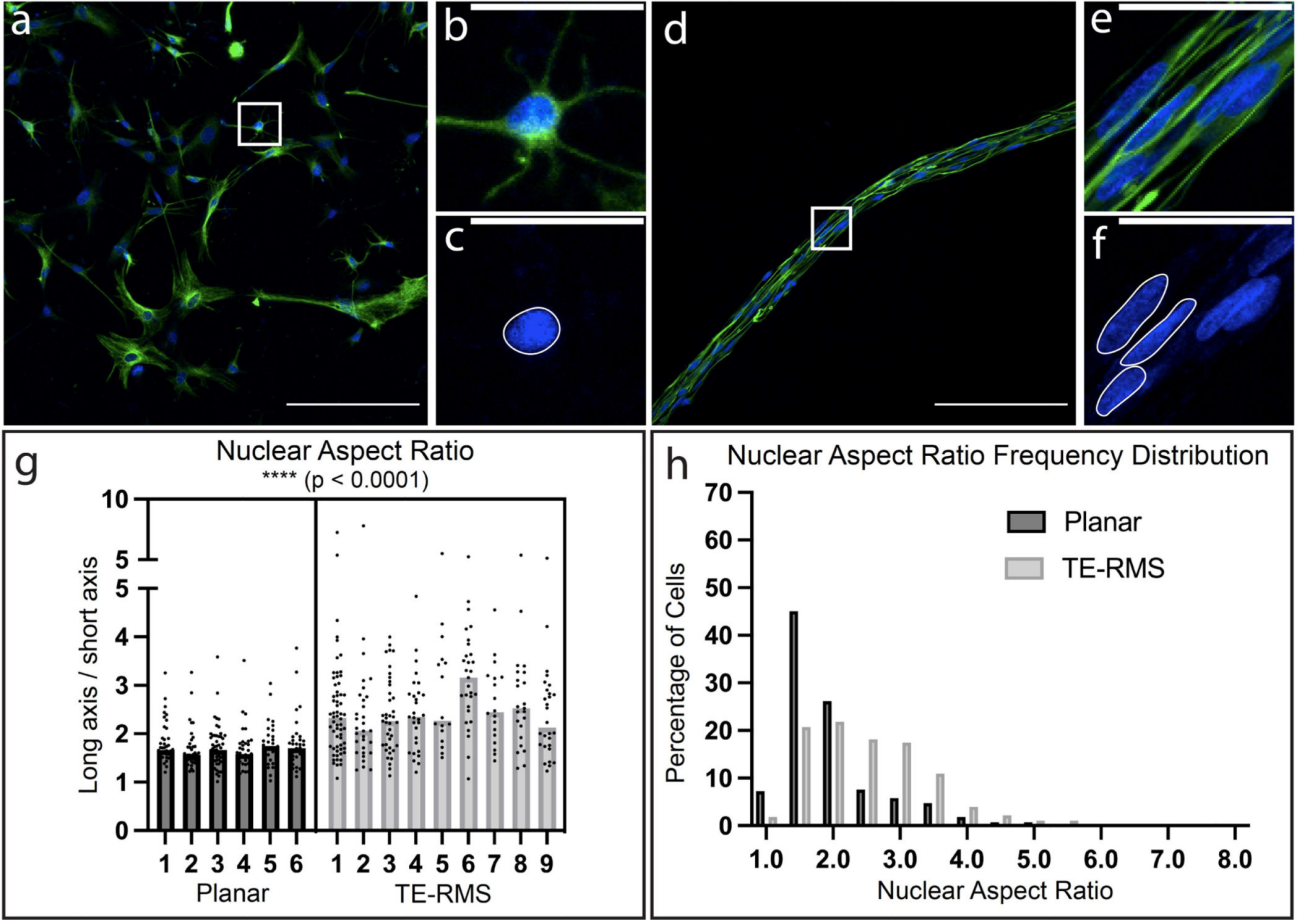
TE-RMS astrocytes have elongated nuclei compared to planar astrocytes. Representative 20× fluorescent image of planar astrocyte culture (**a**) with zoom-in on one single astrocyte depicting nuclear (Hoechst, blue) and cytoskeleton (GFAP, green) labels (**b**) and just nuclear label (**c**). Representative 20× fluorescent image of TE-RMS (**d**) with zoom-in on one single astrocyte depicting nuclear (Hoechst) and cytoskeleton (GFAP) labels (**e**) and just nuclear label (**f**). Example planar nucleus (**c**) and non-overlapping TE-RMS nuclei (**f**) are outlined in white. Nested *t*-test analysis of nuclear aspect ratio of planar astrocytes versus TE-RMS astrocytes (**g**). Each point represents a single cell and bars represent the median of each group. Frequency distribution of the nuclear aspect ratio of planar and TE-RMS astrocytes (**h**). **** *p* < 0.0001. Scale bars: 200 microns (**a**,**d**), 50 microns (**b**,**c**,**e**,**f**).

We performed nuclear measurements in the same planar (Figure 5a–c) and TE-RMS (Figure 5d–f) astrocytes that were used for cytoskeletal analyses described above (Figure 3). The long and short nuclear axes of each cell were measured, and the long nuclear axis was divided by the short nuclear axis to obtain the nuclear aspect ratio for each cell. Nuclear aspect ratios were compared by a two-tailed, nested *t*-test which demonstrated that TE-RMS astrocytes had significantly higher nuclear aspect ratios compared to planar astrocytes (Figure 5g; *t* = 8.041, df = 13, *p* < 0.0001). A higher nuclear aspect ratio equates to a cell possessing a more elongated nucleus. These results indicate that TE-RMS astrocyte nuclei were significantly more elongated in shape whereas planar astrocyte nuclei were rounder. These differences in planar (5c) and TE-RMS (5f) nuclear shape are apparent even in the absence of quantification. Nuclear aspect ratio frequency distributions were plotted for planar and TE-RMS astrocytes (Figure 5h). Statistics were not run on these distributions, but the evident difference in the shape of the distributions highlights the elongation of astrocyte nuclei in the TE-RMS compared to planar sister cultures. Whereas the majority (>50%) of planar astrocytes have a nuclear aspect ratio at or below 1.5, the majority (>70%) of TE-RMS astrocytes have nuclear aspect ratios greater than 1.5, with some TE-RMS astrocytes having a nuclear aspect ratio as high as 8.

### 3.4. RMS Astrocytes Possess Elongated Nuclei Compared to Protoplasmic Astrocytes in the Endogenous Rat Brain

**Figure 6.**
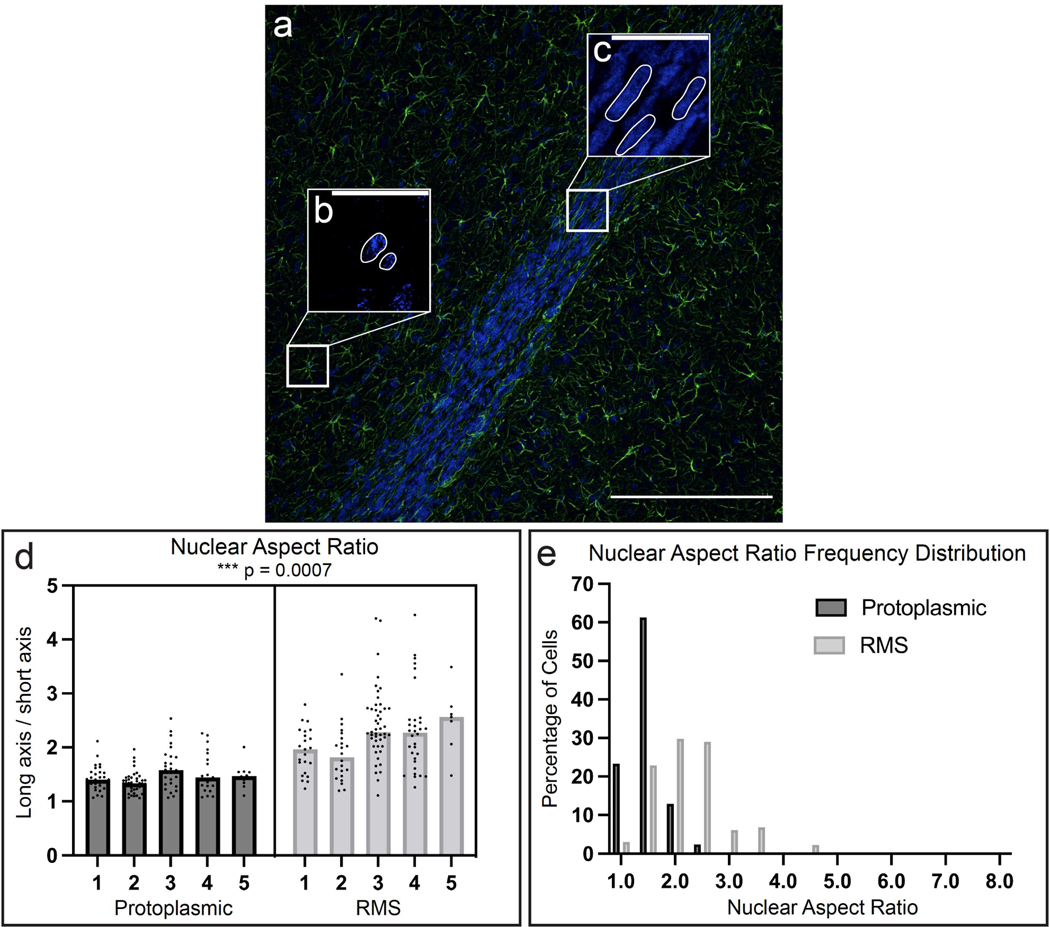
Endogenous RMS astrocytes have elongated nuclei compared to surrounding protoplasmic astrocytes. Representative fluorescent image of a sagittal rat brain slice with nuclear Hoechst stain (blue) and immunostaining for GFAP (green) at 20× magnification (**a**) highlighting the nuclei of a protoplasmic astrocyte (**b**) and several RMS astrocytes (**c**). Example nuclei are outlined in white (**b**,**c**). Nested *t*-test analysis of nuclear aspect ratios of protoplasmic astrocytes versus RMS astrocytes (**d**). Each point represents a single cell and bars represent the median of each group. Frequency distribution of the nuclear aspect ratio of endogenous protoplasmic and RMS astrocytes (**e**). *** *p* = 0.0007. Scale bars: 200 microns (**a**), 40 microns (**b**,**c**).

To compare our in vitro nuclear measurements to astrocytes in the endogenous rat brain, we performed nuclear measurements in the same endogenous protoplasmic and RMS astrocytes (Figure 6a–c) that were used for in vivo cytoskeletal measurements (Figure 4). Nuclear aspect ratios were compared by a two-tailed, nested *t*-test. RMS astrocytes had significantly higher nuclear aspect ratios (i.e., significantly more elongated nuclei) compared to protoplasmic astrocytes (Figure 6d; *t* = 5.372, df = 8, *p* = 0.0007). To further highlight this difference, the nuclear aspect ratio frequency distributions were plotted for protoplasmic and RMS (Figure 6e) astrocytes (statistics were not run on these distributions). Whereas most protoplasmic astrocytes (>60%) had nuclear aspect ratios at or below 1.5 (like in vitro planar astrocytes), most RMS astrocytes (>70%) had nuclear aspect ratios greater than 1.5 (like TE-RMS astrocytes). Additionally, the cell density of the endogenous RMS was so high that it often prevented us from discerning where one nucleus ended and the other began. We measured conservatively and therefore suspect that some endogenous RMS astrocytes have even higher nuclear aspect ratios than reported here.

### 3.5. High Magnification Fluorescent Imaging Highlights Profound Differences in Nuclear Morphology and Cytoskeletal Arrangement between Planar and TE-RMS Astrocytes

**Figure 7.**
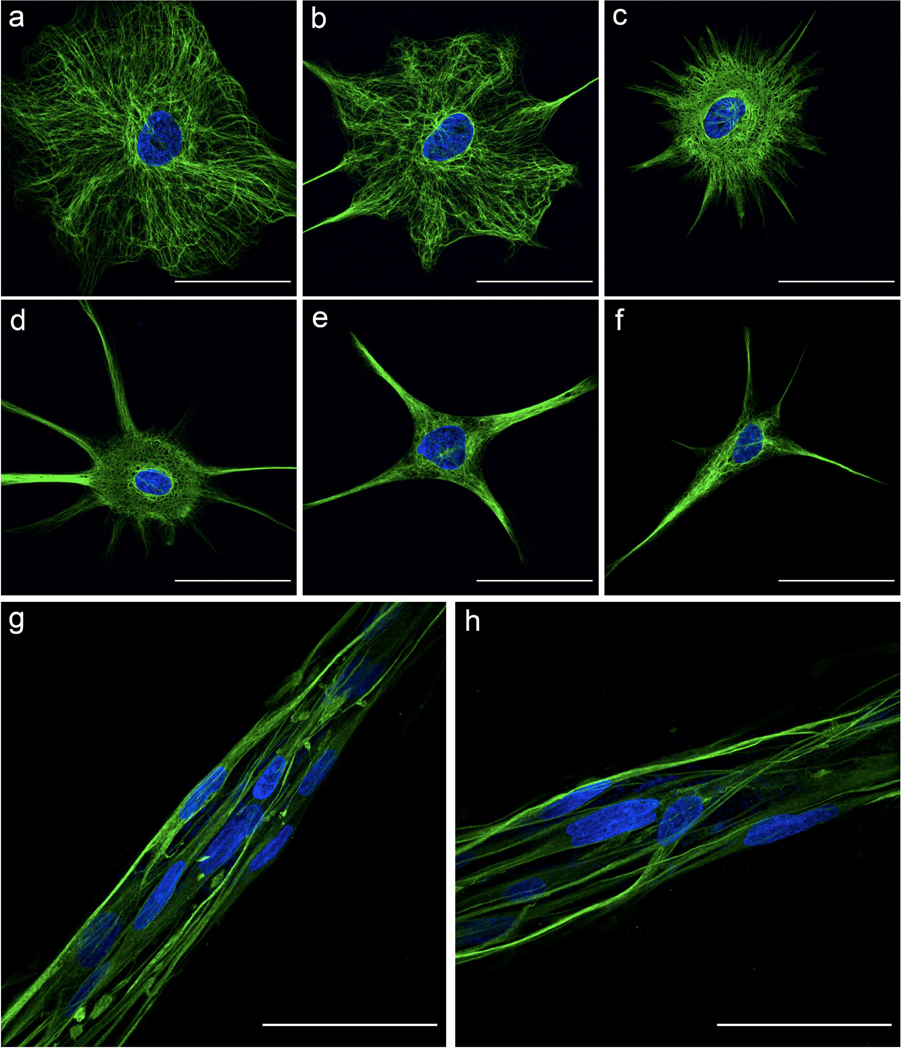
High magnification fluorescent imaging depicting novel astrocyte morphology. High magnification (100×) fluorescent imaging highlighting differences in nuclear shape and intermediate filament arrangement between single planar astrocytes (**a**–**f**) and TE-RMS astrocytes (**g**,**h**). Nuclei (Hoechst) depicted in blue and intermediate filaments (GFAP) in green. All images are compressed confocal z-stacks. Scale bars: 50 microns (**a**–**h**).

High magnification (100×) fluorescent confocal imaging was conducted to examine the differences between nuclear shape and intermediate filament arrangement of planar (Figure 7a–f) and TE-RMS (Figure 7g,h) astrocytes in greater detail. Figure 7 depicts the profound nuclear and cytoskeletal differences between astrocytes in these two different in vitro arrangements. Whereas planar astrocytes possess a round or slightly oblong nucleus, most TE-RMS astrocytes possess nuclei that are significantly elongated. Planar astrocytes exhibit complex, diverse arrangement of intermediate filaments. Whereas some planar astrocytes extend their intermediate filaments in numerous directions (Figure 7a,b), other planar astrocytes (Figure 7e,f) extend collections of intermediate filaments in only a few directions. On the other hand, most TE-RMS astrocytes extend intermediate filaments in two directions from opposite ends of the cell running parallel to the long nuclear axis and longitudinal axis of the construct.

### 3.6. Scanning Electron Microscopy Imaging Confirms Novel Astrocytic Morphology of the TE-RMS

**Figure 8.**
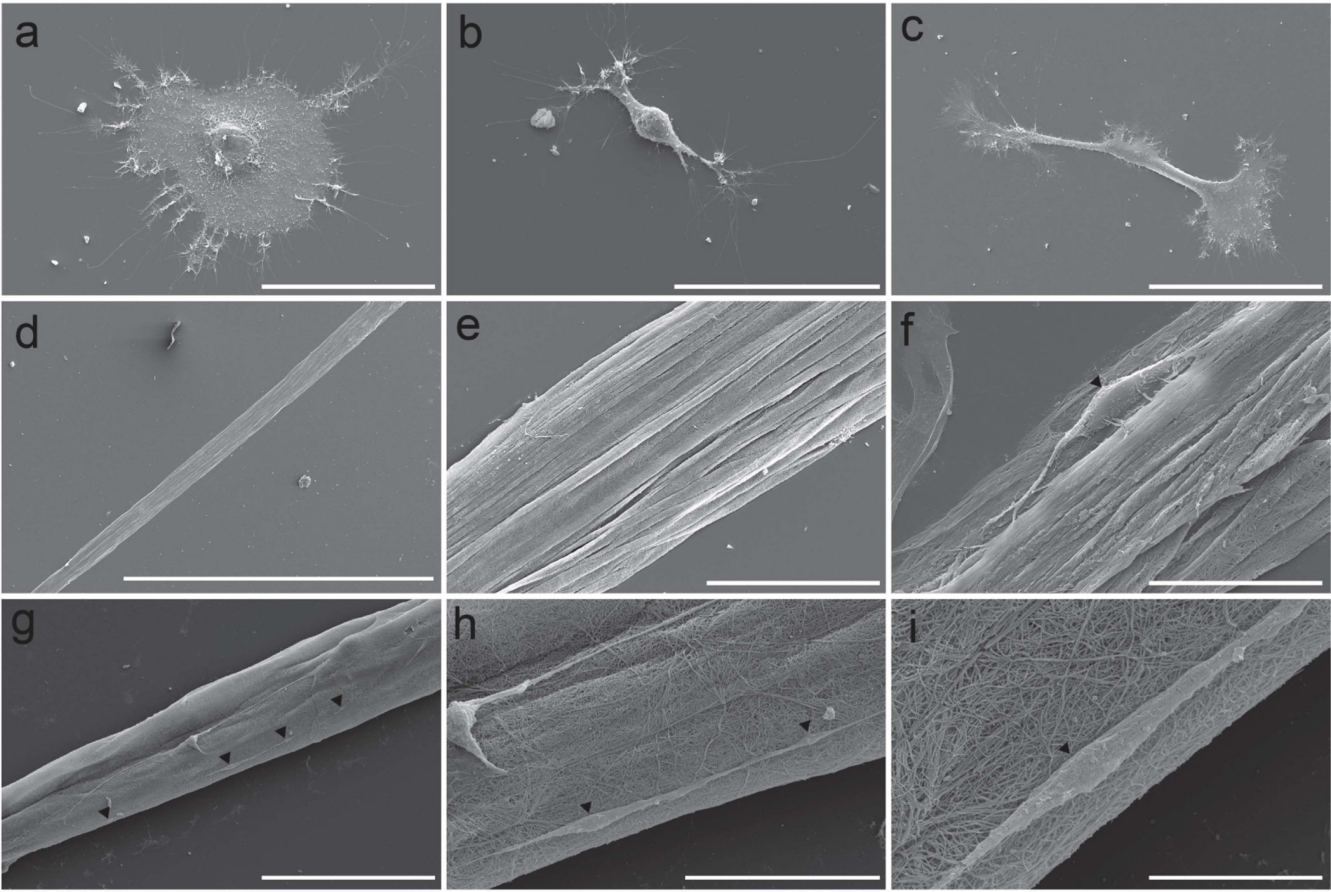
Scanning electron microscopy imaging depicting novel astrocyte morphology. SEM imaging of planar astrocytes (**a**–**c**) and TE-RMSs (**d**–**i**). Single planar astrocytes (**a**–**c**) display process complexity and heterogeneity in morphology. Full length TE-RMS (**d**) with magnified view (**e**) depicting longitudinally aligned, bundled astrocyte processes coated in a fine meshwork of collagen. Single elongated cell with bidirectional processes visible within TE-RMS bundle (**f**). TE-RMS with four distinct astrocytes with visibly connected processes (**g**). Magnified views (**h**,**i**) depicting two cells (**h**) and one cell (**i**) visible on top of collagen network that encompasses TE-RMS construct. Black arrows (**f**–**i**) indicate individual cell bodies visible within the TE-RMS, highlighting their distinct morphology. Scale bars: 10 microns (**i**), 30 microns (**h**), 50 microns (**a**,**b**,**e**,**f**), 100 microns (**c**,**g**), 1 mm (**d**).

To further examine the fine structural differences in nuclear morphology and cytoskeletal arrangement, planar astrocytes (Figure 8a–c) and TE-RMS astrocytes (Figure 8d–i) were imaged via SEM. SEM imaging readily reveals the complexity of processes arising from single astrocytes and the heterogeneity in planar astrocyte morphology. In contrast, TE-RMS astrocytes display bidirectional processes that are longitudinally aligned. Full-length TE-RMS (Figure 8d) with zoom in (Figure 8e) depicts this distinct arrangement of astrocyte processes in the TE-RMS compared to planar astrocytes (Figure 8a–c). Astrocyte bundles (Figure 8e) are coated in a fine collagen meshwork from the polymerized collagen that astrocytes use to pull themselves together during TE-RMS formation. In many cases, the tightly aligned, bundled cell processes and collagen meshwork prevent most single cells from being distinguishable. However, some single TE-RMS astrocytes are visible under SEM (Figure 8f). Some constructs (Figure 8g) reveal several distinct astrocyte somata (black arrows) with visibly connecting processes. Magnified views of these cells (Figure 8h,i) reveal the elongated somata and bidirectional processes of TE-RMS astrocytes as well as the interstitial meshwork of ultra-fine collagen fibrils.

### 3.7. Comparison of TE-RMS and Endogenous Rat RMS Astrocytes

We also directly compared cytoskeletal and nuclear measurements of TE-RMS astrocytes and endogenous RMS astrocytes (data depicted in Table 1). Four two-tailed, nested *t*-tests were performed on measurements of main processes, number of branch points, angle of main processes, and nuclear aspect ratios. To further compare these parameters, we also present descriptive statistics to report the mean and standard deviation (SD). Here, mean ± SD values for TE-RMS and RMS astrocytes were calculated by grouping all cells from each condition (Table 1). Degrees of freedom (DF), t values, and *p* values are from the nested *t*-test analyses run between TE-RMS and RMS groups. This testing showed that the number of main processes (*t* = 0.6326, df = 12, *p* = 0.5388) and the number of branch points (*t* = 0.1509, df = 12, *p* = 0.8825) were statistically equivalent between the TE-RMS and endogenous RMS astrocytes. However, there were modest differences in the angle of the main processes (*t* = 2.861, df = 12, *p* = 0.0143) and the nuclear aspect ratio (*t* = 2.490, df = 12, *p* = 0.0284) between astrocytes in the TE-RMS and astrocytes in the endogenous RMS. Interestingly, these analyses revealed that TE-RMS astrocytes were slightly more aligned and presented more oblong nuclei than endogenous RMS astrocytes.

## 4. Discussion

The TE-RMS is the first engineered biomimetic astrocytic microtissue that is designed to promote sustained neuroblast redirection into distal neuron-deficient brain regions. The TE-RMS may also be a useful platform to investigate the mechanisms of neuronal migration, cell–cell communication, and differentiation in vitro, which may also feature human cells to provide insights into human neurogenesis and migration. The self-assembly of the TE-RMS in biomaterial microcolumns leads to profound morphological changes in astrocytes. We demonstrate that when astrocytes are put into specific 3D conditions with all other conditions kept consistent, they undergo rapid process/cytoskeletal remodeling and nuclear elongation. We have previously demonstrated that a specific angle of curvature of hydrogel microcolumns is required for proper TE-RMS formation [26]. These unique biomaterial conditions initiate a fundamentally different program in these cells that results in dramatic structural changes. These morphological features, which have not previously been described in astrocytes in vitro, do not require growth factors or media changes—rather, they are caused solely by the geometric and topographical feature of the 3D environment that the cells are exposed to. The resulting novel astrocytic phenotype warrants further investigation, for instance as a tool to investigate astrocyte cytoskeletal –nuclear dynamics and cell-environmental interactions that would otherwise not be possible and thereby expanding the capabilities of the TE-RMS as a tool for scientific discovery. Further, we demonstrate that the unique cytoskeletal arrangement and nuclear shape of TE-RMS astrocytes mimics that of astrocytes in the endogenous rat RMS. Whereas planar astrocyte cultures have similar morphology to endogenous protoplasmic astrocytes, the bidirectional processes and elongated nuclei that are characteristic of TE-RMS astrocytes mimic that of RMS astrocytes. These findings augment previous work [29] demonstrating that the TE-RMS mimics the general structure and protein expression of the endogenous RMS, highlighting this technology as the first biomimetic astrocytic microtissue that replicates the structural and functional features of the endogenous pathway for neuroblast delivery in the mature brain.

While the number of main processes and branch points were equivalent between TE-RMS and endogenous RMS astrocytes, we reported small, yet statistically significant, differences in cytoskeletal alignment and nuclear shape measurements between TE-RMS and endogenous RMS astrocytes (as summarized in Table 1). The TE-RMS nuclear aspect ratio of 2.56 ± 0.98 (mean ± SD) was higher compared to that of the endogenous RMS nuclear aspect ratio of 2.23 ± 0.67 (mean ± SD). Additionally, the main processes of TE-RMS astrocytes were more aligned with the longitudinal axes of the cells with a deviation of 12.42 ± 16.42 (mean ± SD) degrees compared to endogenous RMS astrocytes which had a deviation of 18.63 ± 19.39 (mean ± SD) degrees from the longitudinal axes. As these differences highlight, the phenomena of elongated nuclei and aligned cytoskeleton appear to be greater in the TE-RMS compared to that of in vivo RMS astrocytes. There are several possible explanations for these differences. First, our in vitro TE-RMS system is only around 24 h old at the time of these analyses. We predict that this system will continue to mature as it is cultured in vitro to further emulate in vivo RMS morphology. Additionally, our TE-RMS system lacks in vivo cues that could subtly affect astrocyte morphology, including the presence of vasculature and other cell types, the presence of various types of ECM and soluble factors, and signals from migrating neuroblasts. As we continue to advance our TE-RMS system to include these other elements, we will explore their effects on the relationship between TE-RMS and endogenous RMS nuclear and cytoskeletal morphology.

Astrocytes are mechanosensitive, meaning that they can sense and respond to mechanical cues in their surrounding environment [35]. Recent research has begun to elucidate the mechanisms of astrocyte mechanosensation and the significance of this property for physiological astrocyte function in vivo [36,37]. Additionally, nuclear pore complexes, which fuse the two layers of the nuclear envelope, are mechanosensitive thereby making the nucleus itself sensitive to changes in cellular mechanical forces [38–40]. The four main contributing factors to the shape of a cell’s nucleus are cytoskeletal forces, the thickness of the nuclear lamina, level of chromatin compaction, and activity of proteins that control chromatin conformation [41]. Here, we demonstrate significant nuclear elongation of astrocytes following TE-RMS formation, with some astrocytes having a nuclear aspect ratio as high as 8. This extent of TE-RMS nuclear elongation, which has never been reported in planar astrocyte cultures but mimics astrocyte morphology in the endogenous RMS, happens remarkably quickly following astrocyte introduction to 3D conditions. The unique cytoskeletal changes that are concomitant with nuclear elongation during TE-RMS formation indicates that the cytoskeleton is involved in executing these nuclear morphological changes. Cytoskeletal forces propagate to the nucleus through the linker of nucleoskeleton and cytoskeleton (LINC) complex which connects the cytoskeleton to the nuclear envelope [41]. The LINC complex directly contacts cytoskeletal actin filaments and indirectly contacts intermediate filaments through cyto-linker proteins and microtubules through motor proteins to allow for mechanotransduction of extracellular forces to the nucleus [41]. Cytoskeletal forces regulate nuclear size, shape, orientation, and movement within a cell [41]. For example, Versaevel and colleagues demonstrated that the shape and orientation of endothelial cell nuclei can be controlled by compressive forces exerted by actin filaments [42]. Topographical curvature can also control the shape of epithelial cells in vitro, including the orientation and distribution of epithelial cell nuclei and F-actin [43]. Additionally, intra-ocular pressure has been shown to modulate the astrocyte cytoskeleton and nuclear shape in the optic nerve of mice [44]. In the TE-RMS, astrocyte processes align in parallel inside of microcolumns, and they work in conjunction with each other to stretch out the entire cell including the nucleus which can sense these mechanical changes. As cells stretch, the forces acting upon the nuclei become different than the forces acting upon cells in 2D conditions that have radial processes. While we hypothesize that cytoskeletal forces are the predominant mechanism leading to greater nuclear eccentricity, there could be additional factors that are causing these nuclear shape changes. For example, Kalinin and colleagues recently demonstrated that modulation of chromatin compaction with valproic acid treatment leads to changes in astrocyte nuclear shape [45]. We are currently investigating how these biomaterial conditions that induce TE-RMS formation affect the structure of the astrocyte nuclear lamina and chromatin arrangement to elucidate how they may contribute to astrocyte nuclear elongation.

Advanced imaging technologies provide an in-depth view of the astrocyte structural changes that result from TE-RMS formation. High magnification fluorescent imaging (Figure 7) clearly demonstrates the alignment of astrocyte processes and the extent of nuclear elongation that is possible within this biomaterial conformation. The morphological heterogeneity of planar astrocytes is also evident, emphasizing and explaining the variability seen in main processes, branch points, and angle measurements in planar samples. Whereas planar astrocytes are quite morphologically diverse, TE-RMS astrocytes are mostly—but not exclusively—homogeneous in their morphology. SEM imaging (Figure 8) allows us to see the interaction of TE-RMS astrocytes and polymerized collagen that makes formation of these constructs possible. Individual collagen fibrils (which are visible under SEM) have a diameter between 10–500 nm with an average diameter of 40–80 nm [46,47], which is the size of the mesh of small fibers coating the TE-RMS constructs (Figure 8d-i). It appears that the larger bundles (Figure 8e) are astrocyte processes that are bundled and aligned in parallel and coated with this polymerized collagen network. While the specific structure of most cells is obscured beneath this layer of collagen, there are select cells that emerge from underneath the collagen layer and offer a detailed look at TE-RMS astrocyte morphology (Figure 8f–i). The high resolution provided by SEM imaging provides an insightful view of cell–cell contact between TE-RMS astrocytes. The processes of cells are aligned and interconnected to such an extent that it is difficult to discern where one cell ends and another begins (Figure 8h). This starkly contrasts with planar astrocytes that form distinct, discernable domains. Astrocytes engage in cell–cell communication via gap junctions [48,49], whereas other cell types including myoblasts can communicate via cytoplasmic fusion [50]. Although the phenomenon of cytoplasmic fusion has not to our knowledge been reported in astrocytes, advanced imaging techniques utilized here indicate that this phenomenon could be possible in TE-RMS astrocytes. We are currently investigating the potential for astrocyte cytoplasmic fusion upon TE-RMS formation.

Our previous work has begun to elucidate the functional implications of this technology including proof-of-principle evidence that the TE-RMS can direct immature neuronal migration in vitro and in vivo [26,27,29]. In current analyses, we extend these findings to reveal critical details describing the unique astrocyte structure that emerges from exposure to specific 3D biomaterial conditions. We are currently exploring the occurrence of similar structural changes in the TE-RMS fabricated from human astrocyte-like cells, and examining the consequences of these structural changes, including how gene expression regulation changes with TE-RMS formation and the resulting consequences for astrocyte physiology and function. It is well known that mechanical changes in nuclear morphology are often accompanied by changes in chromatin organization and gene expression which then lead to downstream alterations in cell activity and signaling [41,51–54]. However, there is limited research on how mechanically altering nuclear shape affects gene expression regulation and cell behavior in astrocytes specifically [44,45]. Overall, we performed a thorough structural evaluation of the intermediate filament cytoskeleton and nuclear morphology in TE-RMS astrocytes, revealing a novel astrocyte phenotype that has not previously been described in vitro. Current experiments are examining the effects of this morphological rearrangement on astrocyte gene expression, chromatin compaction and organization, organelle structure, and energy dynamics. The TE-RMS offers a powerful platform to investigate critical relationships between biomaterial surface cues and astrocyte behavior and function.

## Figures and Tables

**Table 1.** Quantitative comparison of nuclear shape and cytoskeletal arrangement across TE-RMS and RMS astrocytes.

Nuclear and Cytoskeletal Comparison of TE-RMS and Endogenous Rat RMS Astrocytes					
	TE-RMS Mean ± SD	RMS Mean ± SD	DF	T Value	*p* Value
Number of main processes	2.35 ± 0.69	2.31 ± 0.98	12	0.6326	0.5388
Number of branch points	0.75 ± 0.98	0.78 ± 0.93	12	0.1509	0.8825
Angle of main processes	12.42 ± 16.42	18.63 ± 19.39	12	2.861	0.0143
Nuclear aspect ratio	2.56 ± 0.98	2.23 ± 0.67	12	2.490	0.0284

## Data Availability

The data that support the findings of this study are available from the corresponding authors upon reasonable request. Opinions, interpretations, conclusions, and recommendations are those of the author(s) and are not necessarily endorsed by the National Institutes of Health, the Department of Veterans Affairs, or the Department of Defense.
